# Improving System Integration: The Art and Science of Engaging Small Community Practices in Health System Innovation

**DOI:** 10.1155/2016/5926303

**Published:** 2016-01-24

**Authors:** Pauline Pariser, Laura Pus, Ian Stanaitis, Howard Abrams, Noah Ivers, G. Ross Baker, Elizabeth Lockhart, Gillian Hawker

**Affiliations:** ^1^University Health Network-Toronto General Hospital, Toronto, ON, Canada M5G 2C4; ^2^Department of Family and Community Medicine, University of Toronto, Toronto, ON, Canada M5G 1V7; ^3^Women's College Hospital, Toronto, ON, Canada M5S 1B2; ^4^Division of General Internal Medicine, Department of Medicine, University of Toronto, Toronto, ON, Canada M5S 1A1; ^5^Institute of Health Policy, Management and Evaluation, University of Toronto, Toronto, ON, Canada M5T 3M6; ^6^Department of Medicine, University of Toronto, Toronto, ON, Canada M5S 1A1

## Abstract

This paper focuses on successful engagement strategies in recruiting and retaining primary care physicians (PCPs) in a quality improvement project, as perceived by family physicians in small practices. Sustained physician engagement is critical for quality improvement (QI) aiming to enhance health system integration. Although there is ample literature on engaging physicians in hospital or team-based practice, few reports describe factors influencing engagement of community-based providers practicing with limited administrative support. The PCPs we describe participated in SCOPE: Seamless Care Optimizing the Patient Experience, a QI project designed to support their care of complex patients and reduce both emergency department (ED) visits and inpatient admissions. SCOPE outcome measures will inform subsequent papers. All the 30 participating PCPs completed surveys assessing perceptions regarding the importance of specific engagement strategies. Project team acknowledgement that primary care is challenging and new access to patient resources were the most important factors in generating initial interest in SCOPE. The opportunity to improve patient care via integration with other providers was most important in their commitment to participate, and a positive experience with project personnel was most important in their continued engagement. Our experience suggests that such providers respond well to personalized, repeated, and targeted engagement strategies.

## 1. Background

A high degree of physician engagement is key in successful quality improvement projects aiming to improve health care integration and reduce emergency department (ED) visits and hospital readmissions for individuals with chronic conditions who account for the majority of health care costs [1–7]. Engagement has been defined and operationalized in varying ways. In a review to understand the process of engaging physicians in leadership, Dickinson and Ham found few consistent definitions of engagement and no agreed-upon operational metric by which to measure engagement [1]. Gruen et al. viewed engagement as a social process, defining it as “advocacy for and participation in improving the aspects of communities that affect the health of individuals” [8]. The NHS Alliance notes that engagement is two-way involvement, integral to decision-making, and occurs early in the change management process [1, 3]. In recruitment and engagement of community physicians we emphasize their grassroots involvement to both inform and iteratively improve the key elements of the project [3]. In our context, health care integration has more in common with virtual vertical integration as we sought to establish a clinical network rather than absorb free-standing PCPs into formal organizations [9]. These PCPs have virtual access to services via a centrally coordinated hub and are included in codesigning and developing a shared set of guidelines and protocols [10].

There has been substantial interest in understanding factors that influence physician engagement in improvement initiatives [11–15]. In the primary care field, prior research has identified the following as facilitators of physician engagement: a collegial practice environment, champions and an aligned team, financial resources, knowledge transfer, a grassroots, bottom-up approach to project design and implementation [16–18], and a range of physician characteristics including professional pride and altruism, recognized need for change, and positive attitude [19, 20]. Financial incentives, on their own, have not been found to be sufficient to drive sustained improvements in quality of care [12, 21–23]. Identified barriers to engagement of primary care physicians (PCPs) include limited time and resources, lack of information technology and staff support, and the perception that proposed interventions are either irrelevant or impractical to day-to-day practice [1, 24, 25]. However, these studies have largely focused on barriers and facilitators to PCP engagement within hospital or team/group practice quality initiatives.

Whether these findings can be generalized to engagement of PCPs in small, community-based practices with limited resources, the majority of practicing PCPs in Canada are unclear [26]. There are substantial differences in the capacity of solo PCPs to engage in quality improvement (QI) initiatives; the above-noted facilitators to engagement are relatively less [27–29] and the barriers greater [30, 31]. Thus, small, community-based primary care practices with limited administrative resources may be less amenable to engaging in QI [20, 26], and facilitators to engagement may differ from those of team or hospital-based PCP group practices.

This first paper introduces steps taken to engage community-based solo PCPs in SCOPE: Seamless Care Optimizing the Patient Experience, a project aiming to improve integration of care and to reduce emergency room visits by providing resources to help care for patients with multiple chronic conditions. To evaluate our approach, we examined the perceived importance of various engagement strategies on initial PCP interest and on subsequent PCP participation in the project.

## 2. Methods

### 2.1. Setting

**Figure 1:**
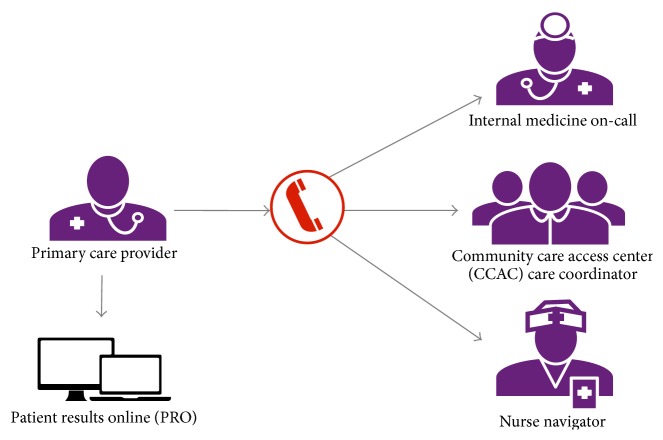
SCOPE provides participating PCPs with a single phone number to access a variety of services.

In the Canadian single payer system, most solo primary care practices function as self-directed autonomous small businesses and cannot be mandated to engage in health system innovations [32–34]. In 2012, we identified a group of community-based PCPs, unaffiliated with any hospital to whom we offered SCOPE resources: a multifaceted intervention that includes access to a nurse navigator, community care resource coordinator, general internal medicine, and online access to hospital-based patient results (Figure 1). The goal of the project was to improve care transitions by formalizing linkages between these doctors' practices and hospital and community resources. Eligible PCPs for SCOPE were community-based family physicians whose practices had high rates of use of the University Health Network (UHN) ED, with limited access to other allied health professionals. Following Gruen et al. [8] and the NHS Alliance [1] we view the engagement of these community physicians as a two-way process, essential to the implementation of the key elements of the project.

### 2.2. Engagement Strategies

This study was reviewed and approved by the institutional research ethics board. A detailed approach was utilized to attract and maintain engagement of the participating PCPs (Table 1). For each PCP, we tracked the number of ED visits and hospital admissions by patients in their practice (identified in the National Ambulatory Care Reporting System database as having the SCOPE PCP as their primary care physician) and contacts with each element of the SCOPE intervention. PCPs with few SCOPE contacts but high practice ED use were targeted for individualized interventions which included personal visits by the primary care lead (PCL) and targeted case consultations by the SCOPE team or a physician champion.

### 2.3. Data Collection

Between February and April 2013, at a nonstandardized time interval during the SCOPE intervention, physicians completed a 15-minute standardized questionnaire by telephone, FAX, or mail. They were asked to provide demographic information about their practice and, from a list of physician engagement strategies and SCOPE project factors, physicians rated the importance of each on a 5-point scale, from “not at all important” (1) to “extremely important” (5), with respect to each of the three engagement phases. This questionnaire surveyed all 3 phases of engagement: initial interest, decision to sign up, and continued participation in SCOPE.

### 2.4. Statistical Analyses

Physician and practice characteristics were summarized as means, medians, or proportions as appropriate. Use of SCOPE services overall and for each component of the intervention was plotted over time. The Spearman correlations between SCOPE use and each of the practice ED visits and hospital admissions for ambulatory care sensitive conditions (ACSCs) were calculated. The number of PCPs with persistently low SCOPE use (lowest two quintiles) but higher practice ED use (upper two quintiles) was examined. For each engagement phase and strategy/project factor, we calculated the mean (standard deviation, SD) PCP importance rating. All analyses were performed using SAS Version 9.2 (SAS Institute Inc., Cary, NC, USA).

## 3. Results

### 3.1. Physician and Practice Characteristics

Of the 50 community-based PCPs whose practices had the highest UHN ED use, 30 consented to participate. Due to project resource and design considerations, participation was limited to 30 physicians with priority given to individuals who either attended an information event or expressed interest but could not attend. Furthermore, simulation studies conducted by our team in preparation for the outcome assessment indicated that a minimum of 25 physicians would provide >85% power to find a clinically important reduction in ED visits over one year (8%). These 30 practices were assessed as being similar to other high use practices by key informants, but there is no data comparing the characteristics of other local high use physicians, so analysis of the differences between these practices and the characteristics of other high use physicians is not possible. All 50 physicians are predominantly older male solo physicians (86%) serving ethnic populations in the local neighborhood. The 50 practices were also similar in their baseline ED visits (see Table 2(b)). Of the 30 PCPs, most were male (83%) and over the age of 50 years (67%) and had been in practice for >15 years (87%). Forty percent reported having >3,000 patients in their practice, with a mean number of patients seen per half-day clinic of 23.4 (range 12–50); 73% used an electronic medical record, 40% used email to communicate with their patients, and 87% provided after-hours services. More than half the physicians (57%) had experience working in an ED (Table 2(a)).

### 3.2. PCP Engagement in SCOPE

**Figure 2:**
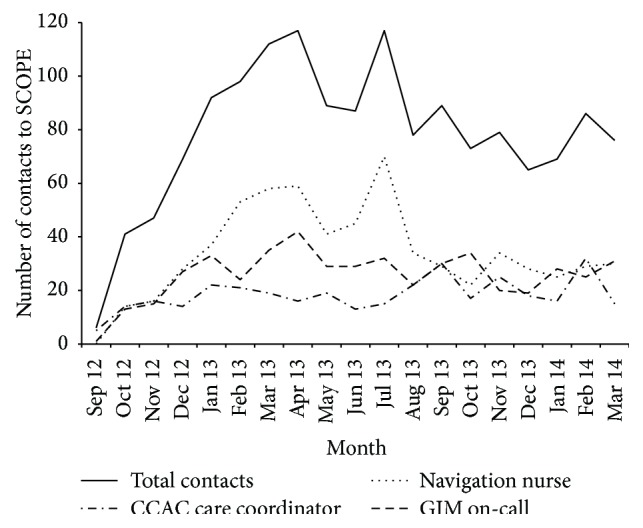
Contacts with individual SCOPE components (September 24, 2012, to March 31, 2014).

The use of the SCOPE intervention components over time is shown in Figure 2 from launch (September 24, 2012) to March 31, 2014. PCP use of SCOPE was positively and significantly correlated with their practice's ED use and rate of hospital admissions for ACSCs (Spearman rho 0.48, *p* = 0.008 and 0.40, *p* = 0.03, resp.). Of the thirty PCPs, only four (13%) had both persistently high practice ED use and low use of SCOPE, as we defined them.

### 3.3. PCPs Perceptions of the Importance of Various Engagement Strategies

#### 3.3.1. Strategies Important in PCPs Initial Interest in SCOPE

PCPs rated acknowledgment by the project team of the challenges of providing primary care in the community of greatest importance in peaking their interest in SCOPE (mean importance rating 4.38/5) (Table 3(a)). All other engagement strategies were seen as moderately important, including the involvement of PCPs as SCOPE team members, recruitment by a PCP in-person, and involvement of hospital administrators (Table 3(a)).

#### 3.3.2. Attendance at In-Person Engagement Events

The relative importance of various factors in determining PCPs' attendance at in-person engagement events is shown in Table 3(b). The time of day the event was held was seen as most important overall (mean importance rating of 3.96/5), followed by the opportunity to network with primary care colleagues and interact with consultant specialists (mean rating 3.81/5 for both). Receipt of continuing education credit for attendance and availability of food at events were seen as relatively less important, with mean importance ratings of 3.05 and 2.64/5, respectively (Table 3(b)).

#### 3.3.3. Decision to “Sign Up for SCOPE”

Physicians indicated that the opportunity to improve care for their patients was the most important factor in their decision to participate in SCOPE (mean importance rating 4.9/5) (Table 3(c)). Enabling access to specific clinical resources, particularly GIM on-call and a nurse navigator, was also seen as very important in PCPs' decision to participate. The opportunities to participate in health system change and improve linkages with local hospitals were also seen as important. Relative to these factors, the opportunity to provide input to the design of the intervention and active inclusion of the clinic front line staff in the recruitment process were seen as only moderately important in their decision to sign up for SCOPE.

#### 3.3.4. Continued Participation in SCOPE

Having positive personal and patient related experiences with SCOPE was seen as most important in PCPs' continued participation in the project (mean importance ratings 4.33/5 for both) (Table 3(d)). The monthly newsletter, in-person events, and project team office visits to the PCPs were all seen as only moderately important to PCPs' continued participation.

## 4. Discussion

Our findings demonstrate that solo PCPs can be effectively engaged in health system integration. However, repeated personalized targeted engagement strategies were required and the most effective engagement strategies varied along the trajectory of the project from recruitment to consent to sustained participation. Health care can be considered a complex adaptive system [35], in such systems that it is often difficult to identify the impact of a single intervention [36, 37]. In rolling out and delivering the SCOPE project, it may have been the interaction of the interventions rather than a specific element per se that influenced the outcomes. It is beyond the scope of this paper to tease out any one element that may have had greater influence.

Our engagement process has elements in common with the concept of developing followership: a social process that emphasizes influencing the behavior of others [38]. The methodology is similar to that of Reinertsen et al. [39], who summarized essential elements to engage physicians in QI initiatives, including agreement on a common purpose, encouraging doctors to become partners, and adopting a transparent communication style.

Acknowledgment by the SCOPE team that primary care of complex medical patients is challenging was the most compelling motivator in the PCPs' decision to learn about SCOPE. The decision to sign up for SCOPE was based on the potential to provide improved care to their patients. This finding is consistent with the broader literature that most physicians' primary focus is with their own practice and the quality of care they deliver [40].

The majority of PCPs used all elements of the intervention to manage their complex medical patients. Further, each of the SCOPE elements received a mean importance rating in the very or extremely important category with respect to PCPs' decision to consent to participate. We believe this reflects that the SCOPE elements were informed by qualitative interviews of PCPs and each component of the intervention was initially vetted with PCPs for potential usefulness prior to launch. Our findings are consistent with that of others which have collectively underscored the importance of a bottom-up approach that ensures the point of view of the clinical provider is incorporated in project design and implementation [13, 41].

PCPs found that the in-person events provided a valuable opportunity for networking with other PCPs and specialist providers and were helpful in improving their linkages with providers and the local hospital. Wagner et al. [42] described this last point as a crucial barrier to overcome in the development of integrated care models. Holding these events at times outside of office hours was essential to PCPs' participation. Our findings support the use of such in-person get-togethers in future quality improvement initiatives.

Achieving an early win with positive personal and patient experiences appeared to be critical to ensuring PCPs' ongoing participation in the project. Such patient centered and provider enhanced foci have been identified as overriding goals in an extensive review of general practitioner perspectives on the management of patients with multimorbidity [43].

In contrast to prior research, our participants did not see a major role for their colleagues as physician champions in motivating their engagement in SCOPE [44]. In health system QI efforts champions typically serve as liaisons, provide leadership and clinical oversight, and secure support for the initiative, particularly from late adopters [45–47]. One potential explanation may be that our PCL was, in fact, a local PCP. Thus, the need for additional “champions” may have been superfluous. Future studies are needed to confirm or refute our findings in the context of primary care initiatives in the community.

A key strength of our study is its focus on understanding what motivates community-based PCPs in small practices to engage in QI initiatives, a topic that has received little attention previously. However, a number of limitations should be kept in mind. First, we assessed impact of engagement strategies only six months after project launch. Thus, the long-term sustainability of PCP engagement is unknown. Second, almost all PCPs were older male physicians with long-standing practices. Data limitations did not permit comparison of those physicians who were recruited to SCOPE with other high ED use physicians in the same community. The extent to which our results may be extrapolated to younger physicians earlier in their careers is unclear. Third, we did not assess for moderating effects of physician and practice characteristics. Effectiveness of physician engagement strategies may differ according to these characteristics. If so, a more tailored approach to PCP engagement accounting for these factors may be required. Finally, we used a very personal and iterative approach to engaging PCPs. This approach required a high level of oversight by the team and PCL, which is resource intensive and may be challenging to scale to other sites. Until further studies can elucidate and confirm high yield engagement strategies that are sustainable, it is unclear whether the methods we have employed are generalizable.

## 5. Conclusion

In spite of a movement toward team-based primary care models, a large proportion of the population is cared for by community-based PCPs in small practices without formal hospital affiliations. These PCPs manage, with limited resources, to develop trusting relationships with patients and provide a compass for their patients' journeys through the health system. Our findings indicate that these physicians can be effectively engaged in quality improvement initiatives, but repeated and targeted engagement strategies are required that incorporate an inclusive style of grassroots consultation, acknowledgment of practice challenges, and provision of interventions that reflect both their expressed needs and their desire to provide excellent patient centered care.

## Figures and Tables

**Table 1:** Physician engagement strategies.

Engagement phase	Strategies
Identification of PCP project lead and physician champions	(i) A primary care lead (PCL), an exemplary practitioner in the target community and recognized leader in system improvement, was chosen for the project. (ii) Among the eligible SCOPE PCPs, five PCPs, whose practices served the major cultural groups in the target community, were identified to act as “champions” by helping to recruit their colleagues.

Initial recruitment	(i) Eligible PCPs were invited to attend one of two in-person events to learn about SCOPE (continuing education credits (MAINPRO-M1) provided). (ii) During the events, participants discussed and prioritized interventions that might help them manage complex medical patients in break-out groups. (iii) Revisions were made to the preliminary SCOPE intervention based on this feedback.

Consent to participate	Office visits by the project coordinator to (i) review the elements of the SCOPE intervention, (ii) complete paperwork and training for the electronic results system, Patient Reports Online (PRO), (iii) provide the contact number for SCOPE resources.

Continued involvement	(i) Regular PCP office visits by the SCOPE clinical team, PCL, and project coordinator for case studies and to inform the PCP of new SCOPE resources. (ii) In-person get-togethers held with participating PCPs and their clinic staff, separately. (iii) A PCP advisory group, comprised of the five physician champions and five PCP SCOPE participants. (iv) Bimonthly newsletter coedited by two volunteer PCPs. Based on ongoing feedback from the various strategies, modifications were made to the intervention and changes were reported to the PCPs.

**(a):** 

Office profile characteristic		*n* (%)
Sex	Male	25 (83.3)
	Female	5 (16.7)

Age	30–39 years	2 (6.7)
	40–49 years	8 (26.7)
	50–59 years	8 (26.7)
	60+ years	12 (40.0)

Years in family practice	≤5 years	1 (3.3)
	6–10 years	1 (3.3)
	11–15 years	2 (6.7)
	>15 years	26 (86.7)

Practice size	≤1000	1 (3.3)
	1001–2000	11 (36.7)
	2001–3000	6 (20.0)
	>3000	12 (40.0)

Hours a week in patient care	30 or fewer	5 (16.7)
	31–40	16 (53.3)
	More than 40	9 (30.0)

Practice appointment scheduling	Same day appointment	24 (80.0)
	Planned appointment (scheduling)	23 (76.7)
	Same day walk-in (no appointment)	17 (56.7)

Uses email to connect with	Patients	12 (40.0)
	Practice team	20 (66.7)

Certification in the College of Family Physicians (CCFP)^*∗*^		17 (56.7)

Experience in the ED (range 1–18 years)		17 (56.7)

Mean number of patients seen in 1/2 day (range 12–50)		23.4

Mean number of hours worked per week (range 24–56)		37.4

Offers after-hours coverage		26 (86.7)

Uses electronic medical record (EMR)		22 (73.3)

Languages spoken by practice staff (other than English)	Portuguese	18 (60.0)
	Italian	13 (43.3)
	Spanish	11 (36.7)
	French	8 (26.7)
	Cantonese	5 (16.7)
	Mandarin	4 (13.3)
	Other	6 (20.0)

^*∗*^Certification in Family Medicine from the College of Family Physicians (CCFP) is the professional designation of the specialty of family medicine in Canada. It is achieved from the College of Family Physicians of Canada (CFPC) after successful completion of both an approved training program and the certification examination.

**(b):** 

	SCOPE PCPs (*n* = 27)	Nonparticipating PCPs (*n* = 23)
Mean	169.4	187.3
Median	145	149
Range	74–444	91–491
Total ED visits	4574	4308

**(a):** 

Engagement strategy	Importance rating (/5)^*∗*^	
	Mean (SD)	Median (range)
The acknowledgment of the challenges of providing primary care in the community (*n* = 29)	4.38 (0.73)	5 (3–5)
Involvement of family practice colleagues as members of the SCOPE team	3.53 (1.04)	4 (1–5)
Initial recruitment by a family physician	3.27 (1.20)	4 (1–5)
Face-to-face recruitment by SCOPE team	3.23 (1.33)	4 (1–5)
Involvement of hospital leaders, for example, CEO (*n* = 29)	3.28 (1.19)	3 (1–5)

^*∗*^Higher importance rating indicates greater perceived importance in influencing PCP decision-making.

**(b):** 

Engagement strategy	Importance rating (/5)	
	Mean (SD)	Median (range)
The time of day the event was held (i.e., evening) (*n* = 25)	3.96 (1.00)	4 (2–5)
The opportunity to network with primary care colleagues	3.81 (0.94)	4 (1–5)
Interaction with specialist colleagues	3.81 (1.02)	4 (1–5)
One-on-one conversation with the SCOPE primary care lead	3.73 (1.12)	4 (1–5)
CME (MAINPRO-C) credit	3.05 (1.43)	3 (1–5)
The availability of food at the event (*n* = 25)	2.64 (1.35)	3 (1–5)

**(c):** 

Engagement strategy	Importance rating (/5)	
	Mean (SD)	Median (range)
Opportunity to provide better care to my patients	4.90 (0.31)	5 (4-5)
Access to clinical resources		
Internist on-call	4.50 (0.73)	5 (3–5)
Nurse navigator	4.43 (0.94)	5 (1–5)
Homecare coordinator	4.33 (0.92)	5 (1–5)
Patient results online (PRO)	4.33 (0.84)	5 (2–5)
Opportunity to participate in health system change	4.43 (0.73)	5 (3–5)
Opportunity to improve linkages to my local hospital	4.37 (0.85)	4 (1–5)
Opportunity to provide input into the intervention	3.84 (1.08)	4 (1–5)
Inclusion of my front line staff in the recruitment process	3.80 (1.19)	4 (1–5)

**(d):** 

Engagement strategy	Importance rating (/5)	
	Mean (SD)	Median (range)
Positive personal experiences with SCOPE	4.33 (0.80)	4.5 (2–5)
Positive experiences for my patients with SCOPE	4.33 (0.80)	4.5 (2–5)
Monthly newsletter	3.73 (0.98)	4 (1–5)
In-person engagement events	3.73 (0.91)	4 (1–5)
Office visits by the SCOPE team	3.67 (1.09)	4 (1–5)
